# Translating and Adapting Research Bioethical Principles to the African Socio-Cultural Contexts: An Integrative Review

**DOI:** 10.13169/ajb.3.1.001

**Published:** 2026-04-08

**Authors:** Veronica P.S. Njie-Carr, Thomas Senghore, Jainaba Sey-Sawo, Effua Usuf, Josette Yeboah, Sheriff Ceesay, Isatou Dampha, Tomilayo Felicity Omotosho, Fatoumatta Jitteh, Jarra Marega, Ramatoulie E. Janha, Leslien Osei Sarpong, Adolf Kofi Awua, Ayodele S. Jegede, Ebunoluwa O. Oduwole

**Affiliations:** 1 University of Maryland School of Nursing, Baltimore, Maryland, USA.; 2 Department of Nursing and Reproductive Health, School of Medicine & Allied Health Sciences, University of the Gambia, Banjul Campus, The Gambia.; 3 Medical Research Council Unit The Gambia at the London School of Hygiene and Tropical Medicine, Atlantic Boulevard, Fajara, Kanifing Municipality, The Gambia; 4 Cellular and Clinical Research Centre, Radiological and Medical Sciences Research Institute of the Ghana Atomic Energy Commission, Accra, Ghana.; 5 Department of Sociology, University of Ibadan, Ibadan, Nigeria.; 6 Department of Philosophy, Olabisi Onabanjo University, Ago-Iwoye, Ogun State, Nigeria.

**Keywords:** African Bioethics, Cultural Sensitivity, Bioethical Principles, Research Ethical Frameworks and Models

## Abstract

Despite growing interest in African bioethics, aligning it with Western bioethical principles remains challenging. This integrated review aimed to develop adaptable research bioethics for African socio-cultural contexts. A comprehensive search yielded 7,175 articles of which 68 were selected for analysis. Only three studies analyzed a framework, but none was adapted in the African socio-cultural contexts. From the 68 papers, six themes emerged, leading to the development of the Socio-Cultural Research Ethics Conceptual Model (SCREM). Translating bioethical principles in Africa is essential for culturally sensitive, inclusive, and equitable research. The SCREM framework is a significant and timely contribution to African bioethics, offering a novel, flexible, context-sensitive approach relevant and applicable to the realities of Africa’s diversity. Future research will validate and refine SCREM to address the unique health research ethical challenges in Africa. This work highlights the importance of culturally tailored bioethics in advancing ethical research in Africa and could profoundly impact bioethics research in Africa and globally.

## Introduction

The field of bioethics has evolved since it was originally conceptualized by Fahr, a German Pastor, philosopher, and educator in 1927 (do Céu Patrão Neves, 2021; Potter, 1971; Sass, 2007) and in 1971 by the American biochemist, Van Rensselaer Potter (Kushe et al., 2009; Pessini, 2013). Bioethics has its roots in the global West and extends to multiple disciplines in modern bioethics (Fayemi et al., 2016; Steinberg, 1995). Modern research ethics frameworks such as the Nuremberg Code, the Declaration of Helsinki, Council for International Organizations of Medical Sciences (CIOMS) Guidelines, and the United States (U.S.) Common Rule, emerged in response to historical abuses in biomedical research. These frameworks are predominantly shaped by perspectives and institutions in the global North (Fayemi et al., 2016; Hyder et al., 2013) and are widely adapted across different settings. The foundational principles often used in these frameworks, referred to as “Western bioethical principles,” such as autonomy, beneficence, non-maleficence, and justice, are rooted in Western moral and philosophical traditions (Beauchamp et al., 1979; Sweet, 2013).

While these principles have provided a strong ethical foundation globally, their implementation in non-Western contexts, particularly in the African socio-cultural contexts, raises critical ethical questions in terms of relevance and inclusivity (Atuire et al., 2020; Jecker & Atuire, 2022). In many African contexts, however, ethical decision-making is deeply embedded in communal values, relational personhood, spirituality, and respect for social harmony. The predominance of Western-centric frameworks, which highlights an individual’s right of choice with utilitarian consideration, has therefore created an ethical gap. This gap reflects a misalignment between universal ethical prescriptions and the lived moral realities of African societies, thus neglecting local cultural values, worldviews, and moral systems. Consequently, leading to limited cultural relevance, reduced community trust, ethical oversight practices, and limited legitimacy within African contexts that inadequately capture local conceptions of responsibility, consent, and well-being. Addressing this gap is critical for developing contextually grounded research ethics frameworks that are both globally informed and locally meaningful.

Across Africa, the bioethics discipline is still evolving, and there is growing scholarly inquiry on how best to integrate African socio-cultural realities into modern research ethical frameworks and principles. While acknowledging Africa’s immense cultural, linguistic, and historical diversities, ethicists and research scholars argue that certain values, such as communitarianism and relational personhood, are common across African societies. To this end, these shared values provide a coherent philosophical foundation for contextualizing bioethics in the African context (Andoh, 2013; Behrens, 2013; Ssebunnya, 2017). However, based on these sources there is no consensus on how to contextualize the African socio-cultural contexts into the Western bioethical frameworks and principles. While some authors advocate for distinctly African bioethics, others support adapting an existing universal framework to better reflect African values (Fayemi et al., 2016). Other authors have debated the future of bioethics in Africa because of the limited practice of bioethics, yet others are calling for a focus on indigenous African bioethics (Ogundiran, 2004; Ssebunnya, 2017).

There is growing and expanding support for African views, values, beliefs, and approaches to be incorporated into current bioethics instead of developing distinct African bioethics (Andoh, 2013). On the contrary, Behrens’s (2013) call for Africa’s version of bioethics suggests a call towards distinct African bioethics. He argued that Africans must be receptive to appeals to their own culture, moral traditions, and ethical values when reflecting on ethical problems and dilemmas to reclaim their dignity and reaffirm their identity. Although these scholars have opposing views, central to their views is the imperative to develop sensitive research ethics that recognize and integrate African socio-cultural values, beliefs, traditions, and norms. Notwithstanding the growing discourse on reflecting African ideologies and traditions in bioethics, the challenge remains in framing African bioethical constructs to align with Western bioethical principles (Barugahare, 2018). Aligning these principles can help balance specificity and universality, drawing on local ethical concepts and promoting an enriched, context-relevant framework (Atuire et al., 2020). For example, in case studies from Ghana, the importance of incorporating African ethical frameworks to reframe ethical dilemmas in healthcare in efforts to offer ethically appropriate tools resonated with stakeholders’ values (Jecker & Atuire, 2022).

Africa is vastly diverse and complex due to its rich cultures, normative values, traditions, languages, phenotypes, and religions. Therefore, contextualization should be based on shared concepts and socio-cultural values that underpin this diversity. Concepts such as community and kinship networks, oral traditions of transmitting knowledge, and elders as repositories of wisdom are common across African cultures. Therefore, African homogeneity, as used in this paper, does not imply a single, uniform culture but rather the shared values and principles underlying the continent’s vast diversity. This supports the call to contextualize bioethics frameworks and principles within the African socio-cultural contexts (Fallah et al., 2025).

To respond to this call, the goal is to develop bioethics rooted in African socio-cultural realities that can be translated and adapted to similar geo-ecosystems. Adapting Western principles involves modifying existing ethical frameworks to fit African contexts while retaining their foundational assumptions. In contrast, developing African bioethics entails constructing ethical systems rooted in indigenous values (which include the socio-cultural themes identified and explicitly addressed in this paper), philosophies, and communal worldviews, offering a genuinely context-driven alternative rather than a derivative adaptation. To better understand bioethics within the African socio-cultural contexts and to contribute to the empirical and conceptual dialogue in African bioethics, we conducted an integrative review to 1) assess the current state of African bioethical frameworks, 2) analyze how Western bioethical principles has been adapted into the continent’s sociocultural contexts, and 3) propose a framework that integrates themes from empirical and theoretical evidence aligned with Western bioethical principles.

## METHODS

### Eligibility, Search Strategy, and Selection Process

We conducted an open literature search with limited filters. Specifically, no date restriction was applied, and the language was restricted to English. The search process was based on the inclusion and exclusion criteria using two academic databases, which were selected with the assistance of a research and education librarian to retrieve the most relevant evidence based on review objectives. The librarian searched Medline through PubMed from 1809, and EMBASE from 1974 to date using specific key and MeSH terms. Details are shown in Appendix A.

### Inclusion and Exclusion Criteria

We based the choice of articles on the following keywords and MeSH terms: Africa OR African “sub-Saharan Africa” OR “sub-Saharan Africa” Indigenous; cross-culture OR cross-cultural adaptation OR translation AND bioethics OR ethics OR “bioethical principles” OR “ethical principles” OR “ethical framework” OR “bioethical framework” OR “conceptual framework.” We excluded conference abstracts, non-peer-reviewed papers, articles that did not mention “bioethics” or ethics, papers not conducted in Africa, and papers not focused on research ethics.

### Source of Evidence Selection

**Figure 1.**
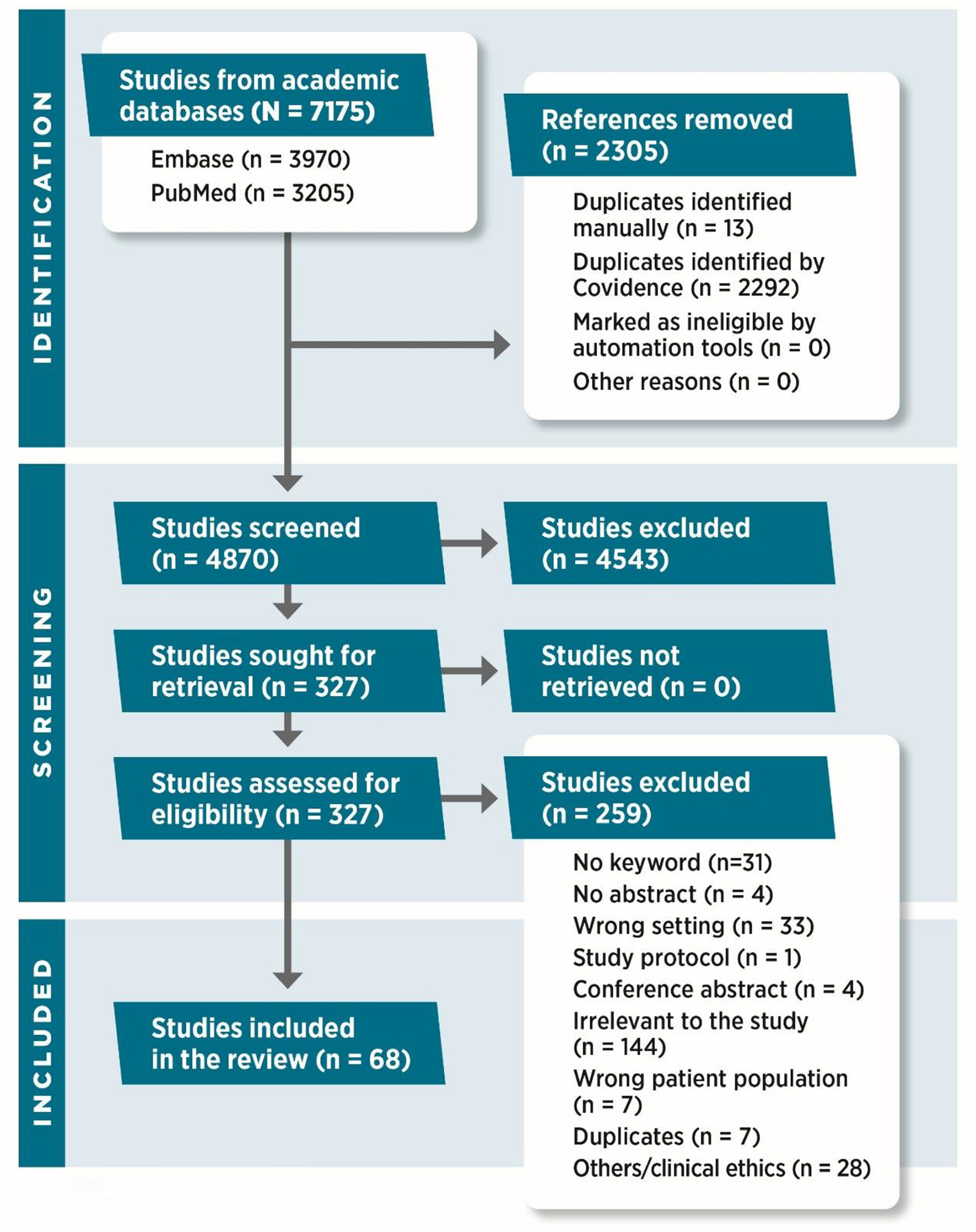
Preferred Reporting Items for Systematic reviews and Meta-Analyses (PRISMA) Flowchart

We retrieved and uploaded 7,175 articles into the Covidence platform (http://www.Covidence.org). Covidence automatically identified 2,292 article duplicates and we manually identified for removal. We developed strategies for each database using pre-defined search terms, incorporating concepts specific to the geographic area of African countries, biomedical research ethics-related issues, and the translation and /or adaptation of bioethical frameworks in the African context (Appendix A). Following the full-text review, we identified 68 papers for analysis that fulfilled the inclusion criteria following the screening process of title and abstract review. See Figure 1.

### Risk of Bias Selection

Authors worked in pairs to review the 68 retrieved papers. The interrater reliability ranged from 0.18 to 0.57. The low IRR reflects the conceptual and contextual complexity of interpreting bioethical principles within diverse African socio-cultural settings, rather than methodological weakness. It can also be justified by the interpretive and context-sensitive nature of qualitative analysis, where raters apply differing lenses shaped by disciplinary backgrounds and career level (including research ethics scholars), conceptual frameworks, or subjective judgments about thematic boundaries. In this study, discrepancies were addressed through a structured consensus process: 1) raters independently reviewed the coding discrepancies, 2) engaged in iterative discussions to clarify interpretations, and 3) referred to the research objectives and codebook definitions to reconcile divergent viewpoints. When disagreements persisted, a third reviewer and/ or the lead investigators adjudicated based on conceptual coherence and fidelity to the data. This rigorous consensus-building process ensured that the final coding reflected shared understanding rather than compromise or bias. Consequently, although the IRR is low, the iterative validation, transparent documentation of decisions, and collective alignment around thematic meanings substantively enhance the credibility, dependability, and overall trustworthiness of the final thematic synthesis. Additionally, we conducted an evidence quality review using the Mixed Methods Appraisal Tool (MMAT) (Pluye et al., 2011). The quality evaluation by the authors showed that all 68 papers were of moderate to high quality.

### Data Coding, Categorization, and Interpretation

Data abstracted were coded systematically using both deductive and inductive approaches. Deductive coding was informed by the review objectives and the bioethical frameworks identified, while inductive coding captured context-specific insights. Coded data were organized into six thematic categories, including Cultural Congruence, Semantic Equivalence/Linguistic Appropriateness, Communitarianism/Collective Responsibility/Pluralism/Interdependence, Cultural Norms, Tradition Preservation, and Infrastructure Development (See Table). These themes informed the interpretation and synthesis of findings, highlighting how research bioethics principles are translated and adapted to align with African socio-cultural contextual realities.

## RESULTS

### Description of the Papers Retrieved

Of the sixty-eight articles identified on the topic of research bioethics, forty-three were non-research and twenty-five were research papers. Qualitative studies were the most prevalent among the research papers, with sixteen publications, followed by seven quantitative research papers, and two mixed-method design papers. Of the sixteen qualitative research papers, thirteen were descriptive studies, and three were case studies.

### State of Science on the Translation and Adaptation of Western Bioethical Principles to the African Socio-Cultural Contexts

In our comprehensive review of the literature on translating and adapting Western bioethical principles within the African socio-cultural contexts, we found a significant gap in the existing literature. Despite the growing discussion on African bioethics, the review showed that no studies reported an explicit translation or adaptation of the Western bioethical principles to align with the African continent’s diverse and unique socio-cultural contexts. However, articles utilized different concepts and principles in the discussion of bioethical issues specific to the African context (Aborigo et al., 2013; Akpa-Inyang & Chima, 2021; Tangwa, 2000; Tosam, 2020) or in the discussion of the unsuitability of the Western bioethical principles to address ethical concerns in the African context (Atuire et al., 2020; Hyder et al., 2013; Mwangi et al., 2022; Sharif & Bugo, 2015). Notably, the qualitative studies primarily focused on descriptive analyses. This analysis thus demonstrates a tendency towards exploratory research rather than the development, translation, and adaptation of frameworks that integrate Western bioethical principles within the socio-cultural African context.

### Level of Translation of the Western Bioethical Principles

Three South African studies addressed bioethical frameworks. Treffry-Goatley et al. (2021) applied the Emanuel Framework to digital storytelling for HIV treatment adherence in rural communities. Lategan et al. (2022) used a Public Health Ethics Framework to assess ethical decision-making in geriatric care across six institutions. Wassenaar and Mamotte (2012) conducted an ethical analysis of eating disorders among students. Other papers highlighted challenges in applying Western bioethical principles in Africa due to cultural diversity and complexity, such as difficulties with informed consent in clinical trials in Nigeria (Aluko-Arowolo et al., 2023). Two studies emphasized the need for bioethics capacity building (Ndebele et al., 2014; Okoye et al., 2017). While authors critiqued Western frameworks, none found adapted them to the African socio-cultural contexts. Appendix B summarizes paper types, contextualized concepts, and Western bioethical principles. This review focuses on translation and adaptation, not comparative analysis.

### Proposed Bioethics Framework Integrating the Western Principles and the Cultural Dimensions Theory into the African Socio-Cultural Contexts

We synthesized empirical and theoretical data from 68 papers and conceptualized six themes: cultural congruence, semantic equivalence/ linguistic appropriateness, communitarianism/collective responsibility/pluralism/ interdependence, cultural norms, tradition preservation, and infrastructure development. Further, we integrated the Cultural Dimensions Theory (Hofstede, 1980) to address the sociocultural context of our proposed framework, consistent with the processes for developing frameworks outlined by Partelow (2023).

### Partelow’s mediating processes for framework development

**Figure 2.**
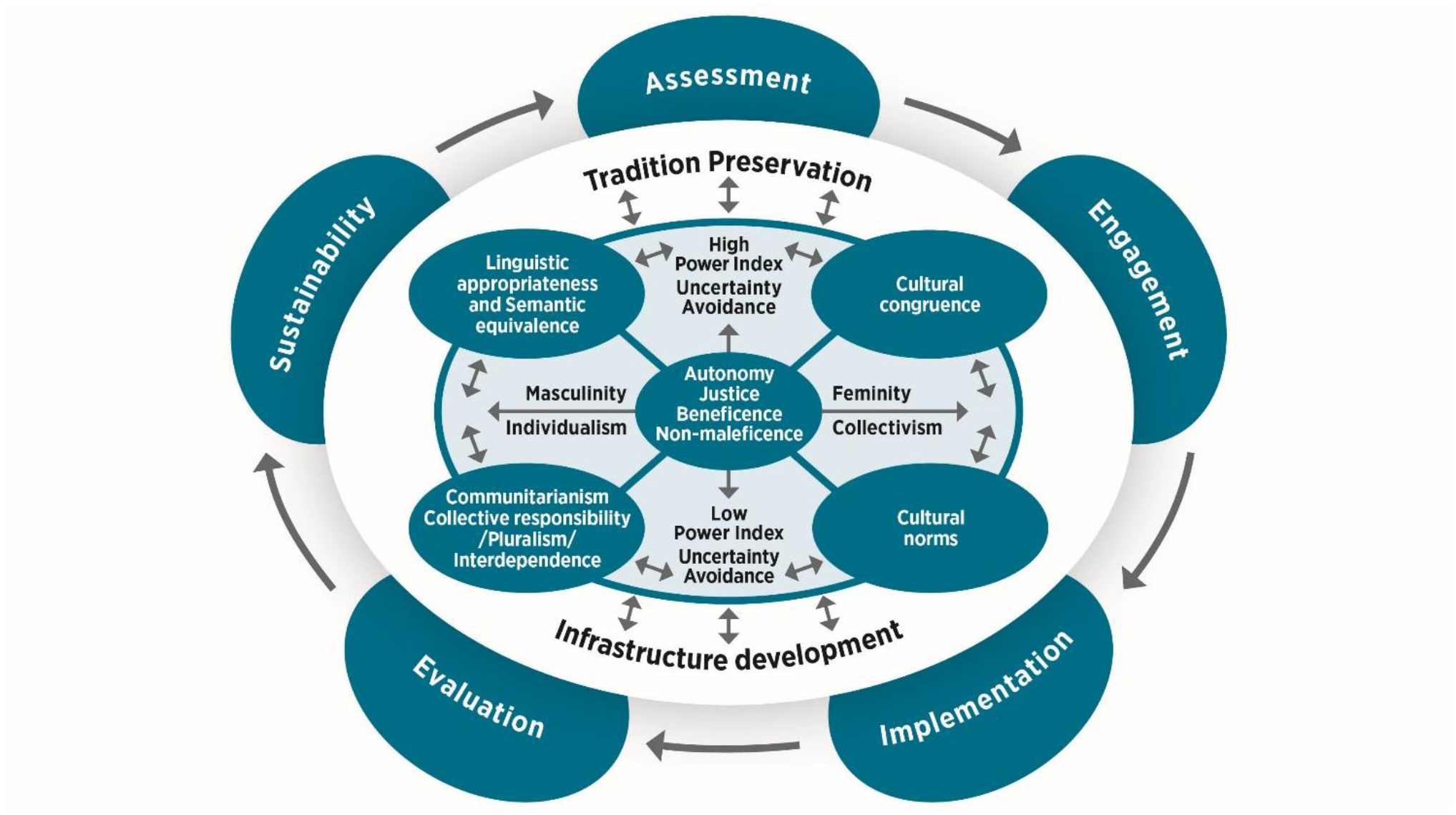
The Socio-Cultural Research Ethics Conceptual Model (SCREM)

According to Partelow (2023), the development and application of frameworks involve “four mediating processes” (p. 6): 1) empirical generalizations, 2) theoretical fitting, 3) application, and 4) hypothesizing. Accordingly, the first step is to align empirical generalizations (identified themes from the literature) with the Cultural Dimensions Theory (socio-cultural context) and the Western bioethical principles (Table). The second step of the development process is theoretical fitting, which requires that the framework proposed is situated within the context of existing theory and principles in the African socio-cultural contexts to fit for purpose. The application and hypothesizing processes highlight utilizing the newly developed framework for study design guidance, ethical review considerations, and testing hypotheses in research studies. Therefore, we integrated the Cultural Dimensions Theory to conceptualize and develop the *Socio-Cultural Research Ethics Conceptual Model (SCREM),* illustrated in Figure 2. The goal is to develop a common language and structure of research bioethics that is malleable and adaptable to Africa’s different socio-cultural variations and tendencies (Behrens, 2013).

### Description of constructs of the Cultural Dimensions Theory

Hofstede’s Cultural Dimensions Theory (CDT) is appropriate and aligns well with the identified themes. The CDT describes society’s cultural values and the interrelationships of the values to human behaviors. Hofstede conducted a population-based survey of 50 countries and initially identified four dimensions on a continuum: Individualism and Collectivism, Power Distance Index, Uncertainty Avoidance, and Masculinity and Femininity. A replication of his study led to the identification of two additional dimensions: Long-term versus Short-term Normative Orientation and Indulgence versus Restraint. Although the CDT has undergone limited validation within African contexts and may not fully capture the complexity and diversity of socio-cultural realities across the continent, the use of the constructs along a continuum is valuable in aligning with Africa’s socio-cultural variations. Moreover, the CDT does not address historical colonialism or the influence of indigenous values, despite that some countries included in its development were formerly colonized. Additionally, while the theory was originally conceptualized to examine workplace behaviors rather than bioethical concerns, its integration into the SCREM framework is justified by its relevance to socio-cultural elements as explicated in the model. Consequently, the CDT represents the most appropriate theoretical foundation to incorporate into SCREM to capture the socio-cultural dimensions.

In our framework, we applied the first four dimensions, which are in approximate alignment with identified themes. The first construct is the *Power Distance Index,* which is the tendency to accept unequal distribution of power and a keen respect for leaders, resulting in high power distance. Low power distance is the tendency for distributive justice and equal power. *Individualism and collectivism* emphasize the self and independence on one end of the continuum, while collectivism prioritizes the notion of “us” and the need to contribute towards a group’s shared goals, thus valuing pluralism, relationships and communitarianism or the idea of harmony morality (Aborigo et al., 2013; Haire & Kaldor, 2013; Metz, 2017; Molyneux et al., 2016). The Ubuntu philosophy, rooted in African humanism, espouses “I am because we are” (Metz, 2011; Metz & Gaie, 2010; Mwesigwa et al., 2023), further underscoring the value of relationships over individual interests and achievements. The *Masculinity and Femininity* construct purports to outline distinct gender roles. The Masculinity dimension is often associated with Western patriarchal systems for men, and the Femininity dimension is associated with culturally prescribed gender roles and socially oriented behaviors for women. *Uncertainty Avoidance* posits that with high uncertainty avoidance, people prefer structures and avoid conflict or risks, and with low uncertainty avoidance, there is a tendency to take risks unbound by structures that are open to conflicts and new experiences.

### The Socio-Cultural Research Ethics Conceptual Model (SCREM)

The SCREM is a flexible high-level theoretical framework that integrates Western bioethical principles (autonomy, justice, beneficence, non-maleficence), themes identified from the integrative review (cultural congruence, semantic equivalence, communitarianism, cultural norms, tradition preservation, infrastructure development), and CDT dimensions (power distance index, uncertainty avoidance, femininity/masculinity, individualism/collectivism) wherein each operates on a continuum and are integrated, allowing context-specific adaptation based on the realities of the different countries and communities. Given Africa’s diverse socio-cultural contexts, it’s impractical to apply the entire SCREM universally and in one study. Instead, using specific components or elements for framework application and hypothesis testing is more appropriate, considering SCREM’s level of theoretical abstraction (Fawcett, 2006).

Although ethics is deeply rooted in Africa’s socio-cultural fabric (Gbadegesin, 1993), bioethics, especially in research, has not been equally developed. We propose placing Western bioethical principles at the core of the SCREM to conceptualize the identified themes. These principles influence six interrelated themes, which vary and, on a spectrum, based on local cultural norms, values, beliefs, and traditions. Positioning Western principles at the core of the model can be critically justified as a pragmatic and epistemically strategic choice rather than an uncritical endorsement of philosophical hegemony. Western bioethical principles such as autonomy, beneficence, non-maleficence, and justice, provide a globally recognized normative foundation that facilitates dialogue, comparability, and legitimacy within global research and policy frameworks. However, their centrality is reframed here not as dominance but as an anchoring reference point, enabling critical engagement and contextual reinterpretation through African moral philosophies such as ubuntu, communalism, and relational personhood. This approach transforms potential hegemony into an opportunity for epistemic negotiation, in which Western bioethical principles serve as a scaffold for intercultural synthesis rather than as a colonizing structure. Consequently, advancing a pluralistic, dialogical bioethics that is both globally intelligible and locally grounded. To align these themes in research bioethics, we consider the Cultural Dimensions Theory constructs (Power Distance Index, Masculinity versus Femininity, Uncertainty Avoidance, Individualism versus Collectivism). SCREM allows for contextual adaptability, integrating Western bioethical principles and Cultural Dimensions Theory in ethical research. See Figure 2.

### The conceptualization of SCREM for pragmatic adequacy

Partelow’s (2023) mediating processes suggest the integration of the Western bioethical principles, the CDT, and identified themes. Consequently, the development and adaptation of the SCREM should be relevant to the unique cultural, social, traditional, and ethical milieu of the specific African communities where research is conducted. Specifically, “taking what is known generally as a guide for what is important to observe” (p.6). We approach this integration through the process of Assessment, Engagement, Implementation, Evaluation, and Sustainability to maximize the utility and pragmatic adequacy of the SCREM Framework.

First, a cultural context *Assessment* is important to lay the foundation for cultural congruence and determine the hierarchical structures ingrained in these local communities. The integration of the *Cultural Dimensions Theory* (CDT) is optimized, focusing on the *High-Power Distance Index*, which amplifies the vital role of elders, religious, and community leaders. Researchers must respect these hierarchies and role designations to engage communities and develop trust. The level of Power Distance Index is tailored to the appropriate context. This is aligned with the expression and preferences of community and religious leaders, impacting the application of the Western notion of *autonomy and justice*. *Collectivism* emphasizes the value African cultures place on *interrelationships, interdependence*, and the idea of *communitarianism and pluralism*. Collective health, well-being, and benefits supersede *individualism* and individual achievements and interests. Collective decision-making with the support of *pluralistic communities* and religious leaders is prioritized, and the need for community consent is respected. *High Uncertainty Avoidance* is prevalent in African countries, and therefore, research designs should be responsive to the level of community concerns, fears, and challenges, and these must be captured for relevance and *tradition preservation*. The informed consent processes (*autonomy*) initiated during *Assessment* should be adapted to the literacy levels and local languages to ensure *semantic equivalence and linguistic appropriateness* to foster self-determination. For example, the strategic utilization of infographics, the visual relay of messages, and scheduling meetings with community leaders and members to explain the research project are integrated into the study protocol. Through interactions with the community, community members’ needs and interests are prioritized in the proposed intervention to minimize potential harm (*non-maleficence*). Notably, the socioeconomic impact of research interventions is also considered as the fair distribution of research benefits, such as opportunities to improve community health within and across different ethnic groups. Consequently, maximizing *Tradition Preservation* through the respect and valuing of *cultural norms* and traditions.

Critical in *Engagement* is the level of community involvement, which is initiated during Assessment by leveraging the perceived *High-Power Distance Index*. Engaging communities and religious leaders early in the research process engenders trust and ensure their input is appropriately incorporated to align with *cultural norms*. During Assessment, researchers would have been informed of the culturally appropriate methods to gather community feedback through individual and community meetings. Consent materials must be culturally relevant and understandable using local language adaptation, including storytelling, and visual illustrations and aids*. Community consent* is essential in the process to ensure that local cultural norms, traditions, power dynamics, and involvement of community leaders are respected. The consent process is validated during Engagement.

*Implementation* is focused on ensuring that culturally sensitive practices through the adaptation of research methods are aligned with local traditions, cultural norms, and the notion of *non-maleficence* to engender cultural humility. Importantly, research staff in partnership with community partners will receive training in cultural sensitivity and humility to enhance *infrastructure development*. For example, cultural events or religious practices are important to understand in the context of research project implementation to promote community decision-making processes and *justice*. Training is tailored to the specific characteristics and practices of communities, community members and leaders. Additionally, community membership on ethical review boards will be invaluable to inform local ethical standards and practices that are respected and adapted to the appropriate *Power Distance Index* level.

*Evaluation* focuses on integrating feedback mechanisms that facilitate ongoing communication channels for individual and collective community feedback on concerns, questions, and suggestions. Mutually agreed upon scheduled meetings, using culturally appropriate methods to collect information, responding to feedback ensuring that community voices are heard and needs responded to promptly for *distributive justice* are appropriate strategies. Another vital component of *Evaluation* is determining research impact on the communities utilizing mixed methods designs and other appropriate metrics, which could be co-designed with community members. Equally important is to share study findings with the communities and ensure leaders have data access to promote equitable access for the promotion of *collective responsibility*.

*Sustainability* focuses on long-term engagement and capacity-building efforts. Long-term engagement requires plans for sustained community involvement beyond the research project. Engaging the community through continued community support and benefits from the research findings is essential. Additionally, researchers are obligated to participate in community-led events and activities that may not be directly related to the research project if sustainability is to be effectuated. Another key component for sustainability is capacity development. Capacity development for *Infrastructure Development* focuses on empowering individuals and communities through training and providing needed resources and tools for local capacity development to support ongoing and future research and related projects. Collaborating with local institutions to ensure the sustainability of research projects and related activities to benefit the community is equally important. Leveraging prescribed gender roles through empowering women to take leadership roles will amplify the *Femininity dimension*. Leveraging the *Masculinity dimension* will support the needs and concerns of women for greater community *autonomy* and mitigate *non-maleficence*.

The SCREM is approached *in continua* consistent with engaging communities to ensure a smooth entry into indigenous groups and long-term sustainability, particularly with the understanding that cultures are not static (Jegede, 2009). The flexibility of the SCREM allows for seamless context-specific adaptation based on the realities of the different countries and communities. For example, a certain point on the power distance index in one community would be different from another although they may ensure remarkably similar extents of tradition preservation. Similarly, when considering gender roles, women could have more autonomy in some communities compared to others. Therefore, the application of the *in continua* approach is critical to the SCREM’s epistemic agility. The intersectionality of the CDT, Western bioethical principles, and empirical/theoretical evidence in the African socio-cultural contexts underscores the importance of culturally informed bioethics that respect and value the application of local contextual realities and practices while adhering to globally held ethical standards for relevance, meaningful impact, and sustainability.

## DISCUSSION

Western bioethical principles guide ethical decision-making globally, but in Africa, adaptation faces challenges due to the diversity in socio-cultural contexts. This integrative review addresses the challenges and proposes the SCREM Framework to bridge the gap between Western bioethical standards and African socio-cultural norms and values. SCREM integrates three components, allowing for a culturally appropriate bioethical framework adaptable to different African contexts. The SCREM illustrates African diversity and complexity but offers adaptable elements to ensure practical application.

The unavailability of a recognized framework designed for African settings limits the ability to effectively resolve ethical dilemmas and lapses because of limitations of the Western bioethical principles (Jecker & Atuire, 2022) in African socio-cultural contexts. Ensuring the social relevance, applicability, and contextualization of ethical frameworks is critically important. Findings from the review support Barugahare’s (2018) concept of bioethics realism, which argues that ethical principles should be pragmatic and relevant within the socio-cultural frameworks of the participants. Some authors (Johnson & Romanis, 2023; Kremling et al., 2023; Metselaar, 2024) suggested that Africa and other regions with similar settings require culturally specific bioethical guidelines. The SCREM provides opportunities for adaptability in research ethics and is contextualized based on socio-cultural realities.

Developing a culturally rooted bioethics framework like the SCREM could profoundly impact bioethics research in Africa and globally. This is particularly relevant when conducting health research in communities where Western decision-making frameworks involve communal or collective consent (Akpa-Inyang & Chima, 2021; Akurugu et al., 2022; Jecker & Atuire, 2022). The reliance on leaders and elders for decision-making aligns with the Power Distance Index from Hofstede’s Cultural Dimension Theory, highlighting a significant contrast with Western bioethical principles, which emphasizes individual autonomy. Our review also highlights the need to balance global research ethical standards with local cultural norms and values, and the increasing focus on relational autonomy (Jecker & Atuire, 2022; Metz, 2017). The SCREM Framework bridges the gap by integrating indigenous values conceptualized in the identified themes and offers a framework for creating an adaptable, culturally sensitive bioethics model for research and a basis to “hypothesize new relationships” (Partelow, 2023, p.6). Future research could evaluate and validate the Model in diverse African communities and cross-cultural research collaborations for its pragmatic adequacy and effectiveness.

Additionally, although SCREM is developed through the lens of research bioethics, it could be applied in clinical ethics and integrated into program curricula to fit for purpose. Consequently, it will promote high-level standards for the responsible conduct of research, research integrity, and rigor for trainees and research scholars in the African context. Institutional review boards and ethics review committees should appropriately integrate the concepts in the review processes to address socio-cultural nuances and not rely entirely on Western bioethical principles. Capacity-development programs are essential through bioethics education, academic training, and mentorship accomplished by formal coursework, informal training, workshops, and ethics review training. Utilizing the SCREM Framework to implement African bioethics from diverse perspective will contribute significantly to the global research ethics ecosystem.

The SCREM Framework is developed to serve as a valuable contribution to the field of African bioethics. We identified five key limitations that warrant discussion. First, our reliance on only two academic databases may have resulted in the omission of relevant literature available in other databases or within the grey literature, which could have provided additional insights. Nevertheless, collaborating with a medical librarian helped to select the most appropriate databases. Additionally, the breadth of our open review yielded substantial evidence, and we observed thematic redundancies that validated our findings. Second, the CDT used to integrate with SCREM was originally developed in non-African contexts. Despite this, the theory remains one of the most comprehensive available and addresses constructs along continua that are highly relevant to African settings. Third, the integration of three distinct components within SCREM may pose challenges to its holistic application. Our intention, however, is to present a flexible and relativist framework that can be adapted to the diverse contexts across the African continent, thereby supporting context-sensitive bioethical health research. Fourth, the diversity and evolving nature of cultural norms across Africa make it difficult to comprehensively address all socio-cultural practices such as rituals and taboos, polygyny, son preference, and inheritance customs. Nonetheless, SCREM’s inherent adaptability allows it to be tailored to specific cultural contexts. Fifth, our analysis was limited to articles published in English, which restricts the generalizability of our findings to socio-cultural contexts in Francophone and Lusophone African countries.

## CONCLUSION

Despite these limitations, this integrative review underscores the need for a contextualized approach to bioethics in Africa, emphasizing the continent’s rich and diverse socio-cultural settings. Translating and adapting bioethical principles in Africa to align with local values and practices is essential for fostering culturally sensitive, inclusive, and equitable research practices. Our analysis reveals significant limitations in applying Western bioethical principles, demonstrating that a universal model is inadequate. To respond to this important gap, the evidence-based SCREM provides a foundational framework tailored to African socio-cultural contexts. This approach paves the way for more appropriate bioethical practices in Africa and similar contexts. The SCREM Framework is a significant and timely contribution to African bioethics, offering a novel, flexible, context-sensitive approach relevant and applicable to the realities of Africa’s diversity. Its adaptability ensures relevance across diverse settings, making it a valuable contribution not only for Africa but also for other regions with similar complex socio-cultural contexts.

## Supplementary Material

1

## Figures and Tables

**Table:** *Alignment of Identified Themes, Cultural Dimensions Theory, and Western Bioethical Principles

Themes Identified from Empirical & Theoretical Evidence	Cultural Dimensions Theory	Western Bioethics Principles
Cultural Congruence	Power Distance Index	Justice Autonomy
Semantic Equivalence /Linguistic Appropriateness	Individualism & Collectivism	Justice Non-maleficence
Communitarianism / Collective Responsibility /Pluralism / Interdependence	Masculinity & Femininity	Autonomy Justice Non-maleficence
Cultural Norms	Uncertainty Avoidance Index	Autonomy
Tradition Preservation		Justice Autonomy
Infrastructure Development		Autonomy Justice Beneficence Non-maleficence

* Alignment utilizing the framework development process by Partelow (2023)

## Data Availability

Data supporting the findings in this manuscript are available upon request.

## APPENDIX A Databases and Search Results

**Table:**

Databases	Search strategies	Number of papers
PubMed (1809-present)	(“Africa” [Mesh] OR Africa[tiab] OR African[tiab] OR “Indigenous Peoples” [Mesh] OR indigenous[tiab] OR Tunisia[tiab] OR morocco[tiab] OR Libya[tiab] OR Egypt[tiab] OR Algeria[tiab] OR Benin[tiab] OR “burkina faso” [tiab] OR “cabo verde” [tiab] OR “cote d’lvoire” [tiab] OR gambia[tiab] OR Ghana[tiab] OR guinea[tiab] OR “guinea Bissau” [tiab] OR Liberia[tiab] OR mali[tiab] OR Mauritania[tiab] OR niger[tiab] OR Nigeria[tiab] OR Senegal[tiab] OR “sierra leone” [tiab] OR togo[tiab] OR zimbabwe[tiab] OR zambia[tiab] OR “south Africa” [tiab] OR namibia[tiab] OR mozambique[tiab] OR malawi[tiab] OR lesotho[tiab] OR eswatini[tiab] OR botswana[tiab] OR angola[tiab] OR uganda[tiab] OR tanzania[tiab] OR sudan[tiab] OR “south sudan” [tiab] OR somalia[tiab] OR seychelles[tiab] OR rwanda[tiab] OR madagascar[tiab] OR kenya[tiab] OR ethiopia[tiab] OR eritrea[tiab] OR djibouti[tiab] OR comoros[tiab] OR burundi[tiab] OR “sao tome and principe” [tiab] OR gabon[tiab] OR “equatorial guinea” [tiab] OR “democratic republic of the congo” [tiab] OR congo[tiab] OR chad[tiab] OR “central African republic” [tiab] OR cameroon[tiab] OR “Sub-Saharan Africa” [tiab] OR “Sub- Sahara” [tiab]) AND (“Bioethics”[Mesh] OR “Bioethical Issues”[MESH] OR “Ethics, Clinical”[Mesh] OR “Biomedical Research/ethics”[Mesh] OR “Ethics, Research”[MeSH] OR “Ethics, Medical”[mesh] OR bioethics[tiab] OR “clinical ethics”[tiab] OR “medical ethics”[tiab] OR “biomedical ethics”[tiab] OR “health ethics”[tiab] OR “bioethical principles”[tiab] OR “ethical principles” OR “bioethical framework”[tiab] OR Theory[tiab] OR “conceptual framework”[tiab]) AND (adapt*[tiab] OR translation[tiab] OR “Delivery of Health Care/ethics”[Mesh] OR implications[tiab] OR “Cultural Competency/ethics”[MAJR] OR “Dissent and Disputes”[Mesh] OR Adopt*[tiab] OR Cross-culture[tiab] OR Tradition*[tiab])	3205
Embase (1974 to present)	(‘Africa’/exp OR ‘Africa south of the Sahara’/exp OR ‘North Africa’/exp OR ‘Indigenous people’/exp OR (africa OR African OR Indigenous OR Tunisia OR morocco OR Libya OR Egypt OR Algeria OR Benin OR ‘burkina faso’ OR ‘cabo verde’ OR ‘cote d’ivoire’ OR gambia OR Ghana OR guinea OR ‘guinea Bissau’ OR Liberia OR mali OR Mauritania OR niger OR Nigeria OR Senegal OR ‘sierra leone’ OR togo OR zimbabwe OR zambia OR ‘south Africa’ OR namibia OR mozambique OR malawi OR lesotho OR eswatini OR botswana OR angola OR uganda OR tanzania OR sudan OR ‘south sudan’ OR somalia OR seychelles OR rwanda OR madagascar OR kenya OR ethiopia OR eritrea OR djibouti OR comoros OR burundi OR ‘sao tome and principe’ OR gabon OR ‘equatorial guinea’ OR ‘democratic republic of the congo’ OR congo OR chad OR ‘central African republic’ OR Cameroon OR ‘Sub-Saharan Africa’ OR ‘Sub- Sahara’):ti,ab) AND (‘bioethics’/exp OR ‘medical ethics’/exp OR ‘research ethics’/exp OR (‘bio-ethics’ OR ‘bioethical issues’ OR ‘bioethics’ OR ‘clinical ethics’ OR ‘medical ethics’ OR ‘research ethics’ OR bioethics OR ‘biomedical ethics’ OR ‘health ethics’ OR ‘bioethical principles’ OR ‘ethical principles’ OR ‘bioethical framework’ OR theory OR ‘conceptual framework’):ti,ab) AND (Conflict/exp OR (adapt* OR translation OR implications OR ‘Cultural Competency’ OR ‘Dissent and Disputes’ OR Adopt* OR ‘Cross-culture’ OR Tradition*):ti,ab)	3970
