# Supplementary material for: Translating and Adapting Research Bioethical Principles to the African Socio-Cultural Contexts: An Integrative Review
Source: Afr J Bioeth. Author manuscript; Available in PMC 2026 Apr 25. (PMC13108696; doi:10.13169/ajb.3.1.001)
Supplement: 1 [file NIHMS2165722-supplement-1.pdf]

Supplementary material

APPENDIX B: *Research and Non-research Papers with Relevant Concepts/Principles*

Author (s) / Year	Country	Design / Sample	Sample size	Contextualized Bioethical Framework / Bioethical Principles / Concepts	Aligned Traditional Bioethical Principles
**Research Papers**					
Aborigo et al. (2013)	Ghana	Qualitative / Community members, physicians, opinion leaders, & field staff	36	Community engagement	Autonomy
Afolabi et al. (2018)	Kenya	Quantitative descriptive / Adolescents, young adults, & parents	235	Comprehension	Autonomy
Akpa-Inyang & Chima (2021)	South Africa	Qualitative / Biomedical scientists & university staff	24	Dominance of African moral philosophies.<br>Communitarianism over individual rights.	Autonomy<br>Justice
Aluko-Arowolo et al. (2023)	Nigeria	Qualitative / Medical practitioners, university lecturers, opinion leaders, traditional health practitioners, & religious leaders	23	Patriarchal influence.<br>Religious dimensions of informed consent.<br>Cultural relativism.	Autonomy<br>Beneficence
De Vries et al. (2014)	Kenya<br>Gambia<br>United Kingdom	Qualitative / Field workers; researchers, ethics committee members, & representatives of funding bodies	49	Potential for harm.<br>Shared morality.<br>Contextual understanding.	Non-maleficence<br>Justice
De Vries et al. (2017)	African countries	Qualitative / Members of ethics committees & national ethics councils	22	Broad consent	Autonomy
Devries, Child, Selbourne, Naker, & Heise (2015)	Uganda	Qualitative / Primary school children	3,700	Violence	Non-maleficence

LICENSE AND COPYRIGHT
© 2026 The Author(s). Published by the Africa Bioethics Network (ABN), in partnership with Pluto Journals in the *African Journal of Bioethics* under the terms of the Creative Commons Attribution 4.0 International License (CC BY 4.0). This license permits unrestricted use, distribution, and reproduction in any medium, provided the original author(s) and source are properly credited. License details: https://creativecommons.org/licenses/by/4.0/ Website: www.africanjournalofbioethics.org

Embleton et al. (2015)	Kenya	Qualitative / Street-connected children & youth (SCCY)	446	Community engagement & equitable participation. Informed consent & assent vulnerability to coercion. Responsibility to protect.	Autonomy Beneficence Justice Non-maleficence
Gumede, Ngwenya, Namukwaya, Bernays, & Seeley (2019)	South Africa	Qualitative / Adolescent-older carer dyads	12	Risk of coercion. Confidentiality.	Autonomy Justice
Hinga, Molyneux, & Marsh (2021)	Sub-Saharan Africa	Qualitative / Research staff	68	Fairness. Obligations of the global community.	Beneficence Justice
Hinga, Marsh, Nyaguara, Wamukoya, & Molyneux (2021)	Kenya	Qualitative / Community members, researchers, & document reviews	115	Emotional and moral distress.	Justice Non-maleficence
Hyder et al. (2013)	Africa Botswana LMIC	Mixed methods / IRB members, research staff, graduate students, researchers	18	Capacity for research ethics in LMICs. Evaluation from internal & external perspectives.	Beneficence Justice
Koloi-Keaikitse, Geller et al. (2021)	Botswana	Qualitative / Paramount chiefs, headmen, elderly persons, traditional healers, youth	48	Community mores. Respect, & respect for gatekeeping as a cultural norm. Culturally informed research regulatory system. Trustworthiness.	Autonomy Beneficence Justice
Lategan et al. (2022)	South Africa (Free State, Northern Cape, and North-West provinces)	Quantitative / Healthcare providers & managers from 6 geriatric institutions	22	Vulnerability. Public health ethics framework. Framework to guide ethical decision-making. Moral dilemmas, prevent future dilemmas and contribute to relationship building. Develop ethical expertise. Promote social justice.	Justice Autonomy.
Leach et al. (1999)	Gambia	Quantitative / Families in urban and rural areas	189	Informed consenting process. Decision of who to consent. Right to make a free choice. Approach to implement informed consent process.	Autonomy Justice

LICENSE AND COPYRIGHT
© 2026 The Author(s). Published by the Africa Bioethics Network (ABN), in partnership with Pluto Journals in the *African Journal of Bioethics* under the terms of the Creative Commons Attribution 4.0 International License (CC BY 4.0). This license permits unrestricted use, distribution, and reproduction in any medium, provided the original author(s) and source are properly credited. License details: https://creativecommons.org/licenses/by/4.0/ Website: www.africanjournalofbioethics.org

Lindegger et al. (2006)	South Africa	Quantitative / Adult patients	59	Understanding components of clinical trials to consent & participate.	Autonomy
Marshall et al. (2006)	Nigeria & United States	Quantitative / Adults	655 individuals (United States: 348; Nigeria: 307)	Informed consent process.<br>Voluntary participation.<br>Study comprehension.	Autonomy
Marshall et al. (2014)	Nigeria	Qualitative / Women	215	Consent process	Autonomy
Meyer, Meyer, Du Toit, et al. (2021)	South Africa	Quantitative / Doctors	3,340	Ethical breaches in confidentiality.<br>Sharing electronic information.	Autonomy
Ndebele et al. (2014)	Malawi	Mixed methods / Clinical trial participants	203	Understanding & awareness of critical trial components.<br>Informed consent process.	Autonomy
Taiwo & Kass (2009)	Nigeria	Quantitative / Research participants from two health centers	113	Informed consent; terminology translation issues	Autonomy
Treffry-Goatley et al. (2021)	South Africa	Qualitative / Participants from local primary healthcare clinics	20	Collaborative partnership.<br>Participatory research.<br>Social value.<br>Scientific validity.<br>Informed consent.	Autonomy<br>Beneficence<br>Justice<br>Non-maleficence
Vischer et al. (2016)	Kenya, Ghana, Burkina Faso, & Senegal	Qualitative / Clinical trial staff	60	Requirements for written & individual consent. Witnesses for vulnerable populations.<br>Voluntary participation.<br>Understanding of the consent process.<br>Adaptation of good clinical practice.	Autonomy<br>Justice<br>Beneficence
Vreeman et al. (2012)	Kenya	Qualitative / Community member assemblies	108	Account for community understanding of research & informed consent.<br>Studies involving children.	Autonomy<br>Justice<br>Beneficence
Wassenaar & Mamotte (2012)	South Africa	Qualitative / Female university students	At least 500	Collaborative partnership.<br>Scientific validity.<br>Social value & social justice.<br>Fair participant selection.<br>Special ethics scrutiny of mental health-related research proposals.<br>Free choice.<br>Coercion because of power dynamics.<br>Risks & benefits.	Autonomy<br>Justice<br>Beneficence

LICENSE AND COPYRIGHT
© 2026 The Author(s). Published by the Africa Bioethics Network (ABN), in partnership with Pluto Journals in the *African Journal of Bioethics* under the terms of the Creative Commons Attribution 4.0 International License (CC BY 4.0). This license permits unrestricted use, distribution, and reproduction in any medium, provided the original author(s) and source are properly credited. License details: https://creativecommons.org/licenses/by/4.0/ Website: www.africanjournalofbioethics.org

				Obligations following the trial.	
**Non-Research Papers**					
**Author (s) / Year**	**Country**	**Topic**	**Focus**	**Contextualized Bioethical Framework / Bioethical Principles / Concepts**	**Aligned Traditional Bioethical Principles**
Akinloye & Truter (2011)	Nigeria	National health policy	Infertility	Rawlsian contractarian principles.<br>Utilitarianism.<br>Quality adjusted life-year (QALY) for ethical review.<br>Ethical dilemma to determining need based on resource constraints.	Justice<br>Beneficence<br>Non-Maleficence<br>Autonomy
Akurugu et al. (2022)	Ghana	Women's autonomy	Marriage, bride wealth, & power	Culturally proper notions of communitarianism - Ubuntu philosophy.<br>Indigenous systems such as the traditional courts.<br>Patriarchal norms.	Autonomy<br>Justice
Atuire et al. (2020)	Ghana	Health policies	Normative framework for healthcare delivery	Respect for people in authority.<br>Relational communitarian morality.<br>Empathic humanism.<br>Harmonious adjustment to relationships.<br>Virtuous character.<br>Social values.	Autonomy<br>Justice<br>Beneficence
Barry (1988)	Developing countries	Human investigation in developing countries.	HIV/AIDS research	Cross-cultural bioethics.<br>Cultural sensitivity & relevance of research ethics.	Autonomy<br>Beneficence<br>Non-maleficence<br>Justice
Bennett & Chanfreau (2005)	Mexico, Senegal Thailand, & Uganda	Rationing antiretroviral therapy	Ethical & equity implications of rationing	Access to treatment.<br>Ethical implications for rationing ART based on compliance.	Justice
Bezuidenhout (2012)	Sub-Saharan Africa	Research infrastructure	Dual-use ethics.<br>Ethics education.	Research oversight.<br>Ethics pedagogy.<br>Notions of research responsibility and precaution.	Non-maleficence<br>Beneficence
Chima, Mduluza, & Kipkemboi (2013)	South Africa	African bioethics agenda	Global ethics.	Philosophy of Ubuntu - we are therefore I am'	Autonomy<br>Justice

LICENSE AND COPYRIGHT
© 2026 The Author(s). Published by the Africa Bioethics Network (ABN), in partnership with Pluto Journals in the *African Journal of Bioethics* under the terms of the Creative Commons Attribution 4.0 International License (CC BY 4.0). This license permits unrestricted use, distribution, and reproduction in any medium, provided the original author(s) and source are properly credited. License details: https://creativecommons.org/licenses/by/4.0/ Website: www.africanjournalofbioethics.org

			Leadership & organizational ethics.	Teaching & learning ethics.<br>Professional ethics.	Beneficence
Choko et al. (2020)	LMIC, Malawi	Ottawa Statement on clinical trials	Ethics of alternative clinical trials group assignment decisions.	Verbal consent.<br>Waive of consent.<br>Ethical decisions for clinical trial intervention & control groups.	Autonomy<br>Justice<br>Beneficence<br>Non-maleficence
Hadler & Rosa (2018)	Uganda	Culturally respective approaches to distributive justice	Cancer patients in limited-resource settings.<br>Global health policy.	Fairness & equitable resource distribution across cultures.<br>Malleable ethics to the different political, economic, power dynamics, & sociocultural contexts.<br>Balancing limited healthcare resources.	Justice<br>Beneficence
Haire & Kaldor (2013)	Uganda	Ethics of HIV treatment as intervention.	Global palliative care	Public health stewardship model.<br>Strict egalitarianism & application to related contexts.<br>Individual autonomy with utilitarian and collectivist concepts of a common good.	Autonomy<br>Justice
Hellsten (2005)	Tanzania	Legal and Ethical concerns in medical research	Bioethics and HIV/AIDS	Global distributive justice and cultural values.<br>Bioethics in the context of HIV.	Justice<br>Autonomy
Jecker & Atuire (2022)	Ghana	Bioethics in Africa	Diversity of cultures.	Ubuntu philosophy.<br>Community.<br>Solidarity.<br>Equality.<br>Dignity.<br>Humility.<br>Consensus building.	Justice<br>Autonomy
Kalabuanga et al. (2016)	Democratic Republic of Congo	Informed consent challenges for vulnerable populations.	Pediatric clinical trials	Respect for persons.<br>Informed consent.<br>Socio-economic vulnerability.<br>Culturally acceptable representative for illiterate participants.	Autonomy<br>Non-maleficence<br>Beneficence<br>Justice
Kamaara, Kong, & Campbell (2020)	Africa	Translation & informed consent	Psychiatric genomic research	Informed consent.<br>Institutional ethics review boards.	Autonomy<br>Non-maleficence<br>Beneficence<br>Justice

LICENSE AND COPYRIGHT
© 2026 The Author(s). Published by the Africa Bioethics Network (ABN), in partnership with Pluto Journals in the *African Journal of Bioethics* under the terms of the Creative Commons Attribution 4.0 International License (CC BY 4.0). This license permits unrestricted use, distribution, and reproduction in any medium, provided the original author(s) and source are properly credited. License details: https://creativecommons.org/licenses/by/4.0/ Website: www.africanjournalofbioethics.org

Kilama (2005)	Africa	Ethics in malaria research	Groups with diminished autonomy (young children, infants & pregnant women)	Human rights.<br>Minimize risks against benefits in mosquito trials.<br>Protection from exploitation.<br>Standard of care.	Autonomy<br>Beneficence<br>Non-maleficience.<br>Justice
Lairumbi, Michael, Fitzpatrick, & English (2011)	Africa	Ethical social value	Research ethics guidelines	Benefit sharing.<br>Promoting social value of global health research.<br>Community partnerships.	Beneficence.<br>Justice
Luseno, Rennie, & Gilbertson (2023)	Sub-Saharan Africa	Review of public health ethics framework	Ethical HIV prevention public health interventions	Social & cultural impacts and unintended consequences.<br>Poor risk/benefit profile.<br>Risk compensation.	Autonomy<br>Beneficence<br>Justice
Mac-Seing, Ringuette, Zinszer, Godard, & Zarowsky (2021)	Uganda	Ethics norms in global health research	People with disabilities & unique needs	Protection of participant privacy.<br>Disability-sensitive data collection.<br>Respectful implementation.	Autonomy<br>Justice<br>Beneficence<br>Non-maleficence
Metz (2010)	Sub Saharan Africa	Bioethics in the African context	Moral theory grounded in indigenous values.	Moral theory grounded on Indigenous.<br>Respect of communal relationships during the informed consent process.	Autonomy<br>Justice
Metz (2017)	Africa	Entrustment to communion	Relational approach to autonomy	Relational approach in African traditions.<br>Ancillary obligations.	Autonomy<br>Non-maleficence<br>Beneficence
Molyneux et al. (2016)	Kenya	Ethics of health systems research.	Context in social relations in the planning & conduct of research	Research ethics framework.<br>Respect to persons & communities beyond the informed consent process.	Autonomy<br>Justice
Moodley & Beyer (2019)	Southern Africa	Ubuntu inspired community engagement model	African philosophy of Ubuntu	Models for community engagement.<br>Responsive communitarianism.<br>Community-based action research.<br>Interconnectedness of humans.<br>Reciprocal relationships.	Autonomy<br>Justice
Mtande et al. (2019)	Malawi	Ethics in clinical trials	Vulnerable populations research	Risk, benefits, & harms.<br>Defining vulnerable populations in resource-limited settings.	Autonomy<br>Beneficence<br>Non-maleficence

LICENSE AND COPYRIGHT
© 2026 The Author(s). Published by the Africa Bioethics Network (ABN), in partnership with Pluto Journals in the *African Journal of Bioethics* under the terms of the Creative Commons Attribution 4.0 International License (CC BY 4.0). This license permits unrestricted use, distribution, and reproduction in any medium, provided the original author(s) and source are properly credited. License details: https://creativecommons.org/licenses/by/4.0/ Website: www.africanjournalofbioethics.org

Mussie, Elger, Kaba, Pageau, & Wienand (2022)	Ethiopia	Bioethical principles for vulnerable populations	Social values & moral underpinnings	Indigenous moral principles.<br>Vulnerable populations in research.	Autonomy<br>Beneficence<br>Non-maleficence
Mwangi et al. (2022)	Tanzania	Bioethics training needs	Postgraduate students, faculty, & researchers.<br>14 online courses; 10-course instructors	Bioethics training in LMIC should focus on the emphasis to adapt to local context.	Beneficence<br>Justice<br>Autonomy<br>Non-maleficence
Owusu (2023)	Ghana	Fair allocation of resources	Children as a vulnerable population	Vulnerable populations & allocation of resources.<br>Delaying vaccine research for children.	Autonomy<br>Beneficence<br>Non-maleficence
Pérez, Hwang, Bygrave, & Venables (2015)	Southern Africa (Malawi, Mozambique, South Africa, & Zimbabwe)	Harm related to SMS (text) messaging	HIV intervention research	SMS service integration<br>Risk-benefit analysis.<br>Respect for persons.	Justice<br>Autonomy<br>Beneficence
Ramabu (2020)	Botswana	Socio-cultural approach to consenting	Children & caregivers.<br>Safeguarding children against sexual abuse.	*Botho* moral approach – respect for persons.<br>Culturally sensitive bioethics.<br>Community leader as part of the consenting process.<br>Situationism Ethical Theory.	Autonomy<br>Beneficence<br>Non-maleficence<br>Justice
Rennie, Gilbertson, Hallfors, & Luseno (2021)	Kenya	Ethics of stigma	Circumcision for HIV prevention among uncircumcised adolescent males.	Social norms of masculinity.<br>Community engagement & mobilization.	Autonomy<br>Justice
Salhia & Olaiya (2020)	Africa	Human participants research and ethics governance	Oncology clinical trials	Communitarianism.<br>Respect for cultures & beliefs.<br>Culture & international ethics.	Autonomy<br>Beneficence<br>Justice<br>Non-maleficence
Sharif & Bugo (2015)	Kenya	Anthropological approach to research bioethics	Patients and their families	Ethno ethics.<br>Decision-making processes.<br>Family dynamics.<br>Social & cultural norms.<br>.	Autonomy<br>Justice<br>Beneficence<br>Non-maleficence

LICENSE AND COPYRIGHT

© 2026 The Author(s). Published by the Africa Bioethics Network (ABN), in partnership with Pluto Journals in the *African Journal of Bioethics* under the terms of the Creative Commons Attribution 4.0 International License (CC BY 4.0). This license permits unrestricted use, distribution, and reproduction in any medium, provided the original author(s) and source are properly credited. License details: https://creativecommons.org/licenses/by/4.0/ Website: www.africanjournalofbioethics.org

Spiegel et al. (2021)	South Africa	Miners assessed for TB and silicosis.	Health equity in implementing artificial intelligence (AI)	Bioethical principles related to AI accuracy, biased training of AI systems, data privacy, capacity development, transparency & accountability, intellectual property ownership.	Beneficence<br>Non-maleficence<br>Autonomy<br>Justice
Ssebunnya (2017)	Africa	Communitarian African bioethics	African bioethicists and philosophers.	African ethno-philosophy.<br>Dominance of principlism.	Justice<br>Autonomy<br>Beneficence
Tangwa (2000)	Cameroon	African philosophy.	African perception of a person.	A “collective mind” in the traditional African approach.<br>Moral responsibility versus moral worth.	Autonomy<br>Justice
Thom (2003)	South Africa	Mental illness, HIV, ethical, and medico-legal implications.	Patients with mental illness and ethical implications for HIV interventions.	Breach of confidentiality.<br>Capacity to give consent.	Autonomy<br>Beneficence<br>Non-maleficence<br>Justice
Thurtle et al. (2021)	Sub-Sahara Africa	Research ethics governance in developing countries.	Regulatory and legal structures.	Strengthening ethics committees.<br>Global health inequalities.	Justice<br>Beneficence<br>Justice<br>Non-maleficence
Tosam (2020)	Africa	Global bioethics	Culturally responsive bioethics	Community/societal determination.<br>Communitarian context.<br>Dignity & human rights.	Autonomy<br>Justice
Van Wyk (2010)	South Africa	Governance infrastructure for research ethics.	Ethical-legal framework.	Research ethics committees.<br>Regulation for clinical trials.	Autonomy<br>Justice<br>Beneficence<br>Non-maleficence
Wareham (2017)	Africa	African-derived moral theories.	Normative ethics.	African metaethics.<br>Influences of power relations, religion, culture, & environment on moral values.<br>Communal values & communal relationships.	Non-maleficence<br>Beneficence<br>Justice<br>Autonomy
Weber, Diop, Gillespie, Ratsifandrihamanana, & Darmstadt (2021)	Senegal	Ethics of imposing practices from Western countries on developing countries.	Developmental needs of children.	Co-develop programs and cooperate with communities.<br>Harness local beliefs and customs.<br>Consideration of local cultures.	Non-maleficence<br>Beneficence<br>Justice

LICENSE AND COPYRIGHT

© 2026 The Author(s). Published by the Africa Bioethics Network (ABN), in partnership with Pluto Journals in the *African Journal of Bioethics* under the terms of the Creative Commons Attribution 4.0 International License (CC BY 4.0). This license permits unrestricted use, distribution, and reproduction in any medium, provided the original author(s) and source are properly credited. License details: https://creativecommons.org/licenses/by/4.0/ Website: www.africanjournalofbioethics.org

					Autonomy
Wilhelmy, Müller, & Gross (2022)	West Africa	Ethical issues during pandemics	Media analysis of published articles on the Ebola epidemic	Responsibility and accountability.<br>Distributive justice.<br>Communication.<br>Research infrastructure.	Justice<br>Autonomy
Zingela, Sokudela, Thungana, & van Wyk (2023)	South Africa	Ethics of genetic counselling.	Patients with schizophrenia	Multicultural & multilingual population groups.<br>Language for communicating informed consent & research-related activities.<br>Distributive justice.<br>Community advisory boards.<br>Build capacity and expertise.	Beneficence<br>Non-maleficence<br>Autonomy<br>Justice
Zuch, Mason-Jones, Mathews, & Henley (2012)	South Africa	Ethical regulatory framework.	School-based adolescent reproductive health research	Adolescent consent.<br>Community consultation for consent.<br>Respect for persons.<br>Governance infrastructure.	Autonomy<br>Justice<br>Beneficence<br>Non-maleficence

LICENSE AND COPYRIGHT
© 2026 The Author(s). Published by the Africa Bioethics Network (ABN), in partnership with Pluto Journals in the *African Journal of Bioethics* under the terms of the Creative Commons Attribution 4.0 International License (CC BY 4.0). This license permits unrestricted use, distribution, and reproduction in any medium, provided the original author(s) and source are properly credited. License details: https://creativecommons.org/licenses/by/4.0/ Website: www.africanjournalofbioethics.org
